# The network structure of self-compassion in older adults with different productive engagement patterns

**DOI:** 10.1038/s41598-025-08157-1

**Published:** 2025-07-01

**Authors:** Huinan Hu, Grand H.-L. Cheng, Stephen Cheong Yu Chan, Eddie S. K. Chong, Peiyi Lu, H. N. Cheung

**Affiliations:** 1 Department of Social Work and Social Administration, The University of Hong Kong, Pok Fu Lam, Hong Kong China; 2 School of Arts and Social Sciences, Hong Kong Metropolitan University, Ho Man Tin, Hong Kong China; 3 Felizberta Lo Padilla Tong School of Social Sciences, Saint Francis University, Tseung Kwan O, Hong Kong China

**Keywords:** Self-compassion, Productive engagement, Older adults, Network analysis, Psychology, Health care

## Abstract

Self-compassion has gained attention as an important factor for mental health. However, research on self-compassion in older adults is limited. This study aims to reveal the interactions among six self-compassion components in older adults and examine how productive engagement influences these components. A total of 807 older adults aged 55 and older in Hong Kong (67% females) participated in the study. They reported their self-compassion and productive engagement through questionnaires. Latent class analysis (LCA) was used to identify different patterns of productive engagement. The EBICglasso method estimated the self-compassion network for the overall sample and for different productive engagement patterns. The LCA results identified a group with low productive engagement (low productive engagement group) and three groups with engagement in certain productive activities (productive engagement group). The network analysis found that *isolation* was the central component in the overall sample and in the low productive engagement group. *Mindfulness* was the central component in the productive engagement group. A dual effect of productive engagement group reported that while it may enhance mindfulness, it might also contribute to the feeling of isolation. Further research is needed to explore these relationships in diverse sociocultural settings.

**Supplementary Information:**

The online version contains supplementary material available at 10.1038/s41598-025-08157-1.

## Introduction

Self-compassion, a positive self-attitude toward suffering and failures, has been identified as a protective factor in improving older individuals’ mental health^1,2^. A large number of studies indicated that self-compassion was related to high levels of positive psychological constructs (e.g., well-being, personal growth) and low levels of psychopathology symptoms (e.g., depression, distress) in older adults^3–6^. However, studies on the structure and development of self-compassion in older adults are still limited, especially for East Asian cultures^7,8^. Understanding the structure of self-compassion and how it may differ in different cultural groups can benefit the interventions aimed at improving older adults’ well-being.

Neff^1^ conceptualized self-compassion as three basic domains, with two opposing components in each domain: (1) being understanding and kind to oneself (*self-kindness* versus *self-judgement*); (2) viewing personal experience as part of common human experiences (*common humanity* versus *isolation*); (3) holding a balanced state about personal suffering feelings and thoughts (*mindfulness* versus *overidentification*). Neff^9^ further integrated them into the bipolar continuum structure ranging from uncompassionate self-responding (UCS: *self-judgement*, *isolation*, *overidentification*) to compassionate self-responding (CS: *self-kindness*, *common humanity*, *mindfulness*). Thus, self-compassion can occupy any position on this continuum, from UCS to CS or the neutral midpoint^10^.

Yet, the continuum and separation of UCS and CS in the construct of self-compassion remain a heated and dominant debate^11^. In other words, increasing CS may not relate to reducing UCS, and individuals can present high UCS and CS (or low UCS and CS) simultaneously^12^. Particularly relevant culturally, individuals in East Asian dialectical cultures (e.g., China and Japan) might be more likely to have independent UCS and CS^12,13^. This is because dialectical culture in the East fosters openness to holding seemingly contradictory traits, and therefore individuals with dialecticism might be easier to accept the coexistence of opposite traits within themselves, including UCS and CS^12–14^. In line with this perspective, the present study measured both UCS and CS to capture the potential distinct structure of self-compassion in Chinese dialectical culture.

Yet the manifestation of self-compassion could be affected by productive engagement. Productive engagement has been a key indicator of gerontology research on successful aging and refers to paid or unpaid activities that generate benefits to both individuals and the society^15–17^. The covering range of productive engagement in Eastern Asia includes activities related to economic and social values (e.g., work, volunteering), as well as activities contributing to the family (e.g., caring for family members)^18,19^.

According to role theory, productive engagement may affect the structure of self-compassion^20,21^. Due to retirement and withdrawal from formal employment, work-related role loss is a common and critical characteristic in individuals’ later life^20^. However, these social roles could be made up through productive activities which could affect self-compassion. The role theory postulates two competing pathways regarding the influence of productive engagement in individuals’ self-compassion: role enhancement and role strain^22,23^. On the one hand, social roles afford older adults access to social connections, life meaning and purpose, which can fulfill their emotional needs and promote their self-worth^15,24^. Emotional gratification and self-worth improvement are reported to be important factors in increasing self-compassion^25,26^. On the other hand, failure to satisfy the role demands might make individuals overwhelmed in productive activities and place them at greater risk of stress^15,24^. Uncompassionate self-responding, such as self-judgement, may occur when older adults experience role strain. Thus, the manifestation of self-compassion might be influenced by the productive engagement of older people.

Notably, previous studies focused on various patterns of different types of productive activities via latent class analysis (LCA)^20,27^. However, engagement in different productive activities might have similar effects on older adults’ mental health^20,27–29^. Work-related (e.g., employment), family-related (e.g., grandchildren caregiving) and community-related (e.g., volunteering) productive activities can provide older adults with a similar sense of life meaning and social links to their colleagues, family members, or neighbors^29,30, ^and similar challenges when older adults try to satisfy role requirements^15^. Besides, the role enhancement argues that engagement in productive activities may facilize the engagement of other activities by providing opportunities to access more social resources^20^. Thus, the engagement of productive activities might be more important than the number and type of productive activities in affecting older adults’ mental health, including their self-compassion. Based on this, patterns with high engagement in some productive activities might differ in self-compassion from the pattern with low engagement in all productive activities. However, the discussion of the relationship between productive engagement and self-compassion in older adults has been lacking.

To fully reveal the structure of self-compassion in older adults, network analysis can be adopted to explore the complex correlations among self-compassion components of older adults with different productive engagement patterns. A network includes nodes (i.e., self-compassion components) connected with edges (i.e., partial correlations between self-compassion components)^31^. Network analysis can identify central nodes and central associations in the network^32^providing insight into the component contributing the most to the self-compassion network. For example, Zhao, et al.^33^ conducted the network of self-compassion in Chinese adolescents and found that *mindfulness* might exert the most core role in the self-compassion network, which can be focused on to benefit adolescents’ self-compassion. Also, given the focus on self-compassion in psychotherapeutic interventions, an in-depth understanding of its structure and relatively central component could help practitioners to identify crucial components and better apply relevant interventions to improve clients’ mental health^6,34,35^. For example, if the mindfulness is identified as the core component in older adults’ self-compassion network, practitioners can focus more on the improvement of mindfulness. However, while most studies focus on younger populations, there is a dearth of research on the network structure of self-compassion and its association with productive engagement in Chinese older adults, which needs more exploration.

Therefore, this study aimed to reveal the unique relationships among self-compassion components in Chinese context by exploring the network structure of self-compassion in Chinese older adults, as well as comparing the network structure of self-compassion in older people between the productive engagement pattern and the low productive engagement pattern.

## Methods

### Ethics statement

This study was approved by the Ethics Committee of Hong Kong Metropolitan University (Number: HE-RGC2020/AS02) and performed in line with the principles of the Declaration of Helsinki. All participants provided informed consent.

### Participants and procedures

This study was based on a Hong Kong government-funded project aiming to promote older adults’ well-being in Hong Kong from 2021 to 2022. Based on the search about Active Ageing Index by São José, et al.^36, ^the sample in this study was restricted to adults who were aged 55 or above. Potential participants in this project were recruited from local communities via online advertisements, public fliers, the university’s aging register, and previous participant information. Only those who provided informed consent can participate in this study. Participants were invited to complete questionnaires via the online link or paper survey.

Given that primary measures of the study’s interesting variables were not assessed in the first wave data, the current study only used the second wave data of this project which was collected in 2022. The final sample included 807 participants. Most participants were females (67%), aged 55–59 (33.3%), unemployed/retired (63.4%), married (63.6%), and had no religion (62.9%). About 28% of participants had tertiary education and above. About 55% of participants had a family monthly income below 30,000 HKD. Demographic information is presented in Table 1.

**Table 1:** Participants’ demographic information (*n* = 807).

Characteristics	*n*	%
Gender		
Male	266	33.0%
Female	541	67.0%
Age		
55–59	269	33.3%
60–64	229	28.4%
65–69	154	19.1%
70–74	92	11.4%
75–79	38	4.7%
80 and above	25	3.1%
Education level		
Primary school or below	102	12.6%
Junior secondary school	160	19.8%
Senior secondary school	322	39.9%
Tertiary	223	27.6%
Family monthly income		
Below 10,000	181	22.4%
10,000–19,999	153	19.0%
20,000–29,999	108	13.4%
30,000–39,999	64	7.9%
4,000–49,999	42	5.2%
5,000 or above	64	7.9%
Missing data	195	24.2%
Marital status		
Married	513	63.6%
Unmarried	294	36.4%
Religion		
Yes	299	37.1%
No	508	62.9%

### Measures

#### Productive engagement

Indicators used to assess older adults’ productive engagement were adapted from the research by Cheng, et al.^24,^ including 8 productive activities: employment status (working for pay), life-long learning (taking any courses or training), formal volunteering (providing unpaid assistance to any organizations or groups), informal volunteering (providing unpaid assistance to friends, neighbors or other non-relatives), housework support (providing housework to any family member), spouse caregiving (caring for their spouse), grandchildren caregiving (caring for their grandchildren), and parents caregiving (caring for their parents). Participants reported their engagement in these eight activities over the past 12 months, and their responses to each activity were coded as “0” or “1”. For employment status and life-long learning, participants were asked to report *yes* (coded as “1”) or *no* (coded as “0”), because these activities typically involve a single and overarching status (e.g., working vs. not working, enrolling vs. not enrolling in any course); for formal volunteering, informal volunteering, housework support, spouse caregiving, grandchildren caregiving, and parents caregiving, participants were asked about their weekly frequency of engagement in these activities (*less than once a week* was coded as “0”, *at least once a week* was coded as “1”), because these activities are frequent and require individuals’ sustained involvement^24^.

#### Self-compassion

The Self-Compassion Scale-Short Form (SCS-SF) developed by Raes, et al.^37^ was applied to measure older adults’ self-compassion. The 12-item SCS-SF consists of six dimensions: self-kindness (e.g., I try to be understanding and patient towards those aspects of my personality I don’t like), self-judgement (e.g., I’m disapproving and judgmental about my own flaws and inadequacies), mindfulness (e.g., When something painful happens I try to take a balanced view of the situation), over-identification (e.g., When I’m feeling down I tend to obsess and fixate on everything that’s wrong), common humanity (e.g., I try to see my failings as part of the human condition), and isolation (e.g., When I’m feeling down, I tend to feel like most other people are probably happier than I am)^37^. Each item was scored on a 5-point scale ranging from 1(not at all) to 5 (always). The Chinese version of the questionnaire was translated and back translated by the research team^38^. Previous research proved the good validity and reliability of this scale in Hong Kong older adults^39^. The Cronbach’s alpha coefficient of this scale in the current study was 0.87, and the confirmatory factor analysis showed a good fit of the current sample to the original six-factor structure (*χ*^2^*/df* = 2.81; RMSEA = 0.047, CFI = 0.99, TLI = 0.98, SRMR = 0.036).

### Data analysis

The data analysis was conducted in SPSS 25.0, Mplus 8.8, and R 4.3.1. The SPSS 25.0 was used to conduct descriptive statistics among self-compassion dimensions and productive engagement items (results are shown in Supplementary Table S1). Then, Mplus 8.8 was used to build latent class analysis (LCA) models to classify the patterns of productive engagement^40^. A series of models were examined to find the optimal model. The following indices were used to guide the optimal model: the Akaike Information Criterion (AIC), Bayesian Information Criterion (BIC), and sample-size adjusted BIC (aBIC), with lower values indicating a better model fit^41,42^; the Lo-Mendell-Rubin test (LRT) and bootstrap likelihood ratio test (BLRT) with significant *p*-values indicating that the model of K classes has a better fit than the model of K-1 classes^43^; and entropy with a value above 0.6 suggesting an acceptable model^44^.

Based on LCA results, network analysis was conducted using R 4.3.1 to explore self-compassion network structure in older adults as well as the association between the self-compassion network structure and productive engagement.

#### Pre-analysis

Redundant nodes in the network might lead to biased results. Therefore, the goldbricker function in the R package *Networktools* was used to check the potential redundancy of nodes^45^. Collinearity was identified between *overidentification* and *isolation*. However, due to their nature as the core constructs of self-compassion, the two nodes were still included in the subsequent network analysis.

#### Network analysis

The EBICglasso function in the R package was used to estimate the network model^46^. The package used the graphical LASSO function combined with the extended Bayesian information criterion (EBIC) to construct a graphical Gaussian model (GGM)^47,48^. The EBICglasso was selected for the network modeling because it was found suitable for conditions similar to our study (sample size, continuous data), especially in performing excellent sensitivity, consistency between true and estimated edge-weights and consistency between true and estimated node centrality, which indicated a reliable estimation of the centrality^49–51^. In the network, each node represents a component or symptom, while edges visualize associations between components or symptoms. Wider edges indicate stronger correlations between nodes, with green edges representing positive correlations between nodes and red edges representing negative correlations. Expected influence and strength were calculated to assess the importance of a node in the network. Expected influence is the sum of edge weights (both positive and negative) for a node with other nodes^52^. Strength is the sum of all absolute weights of the edges for a node with other nodes^53^. A node with high expected influence and strength might be strongly connected to other nodes and be a potential central symptom. However, Spiller, et al.^54^ reported that only expected influence successfully predicted the degree to associations between changes in nodes and changes in other nodes. Therefore, we mostly took reference to expected influence in this study to identify the central node.

The 95% confidence intervals (CIs) with bootstrap were applied to evaluate the accuracy of edges, and the correlation stability coefficient (CS-coefficient) was used to evaluate the network stability. A CS value is ideally higher than 0.5 and a CS value exceeding 0.7 indicates high stability^46^.

Finally, to further examine the association between self-compassion network structures and productive engagement, the self-compassion network structures between different productive engagement groups were compared.

#### Comparison of networks

The R package *Network Comparison Test* was used to evaluate the global strength invariance and network structure invariance of the two cross-sectional networks^55^. Centrality invariance tests for each node and edge-wight difference tests for each edge were also conducted.

## Results

### Groups of productive engagement in older adults

LCA identified four classes, namely “family engagement group” (33.0%; average “caregiving for spouse” and “housework support children”), “working-family engagement group” (17.1%; average “caregiving for spouse”, high “working” and “housework support children”), “society-family engagement group” (6.6%; high “volunteering”, average “learning” and “caregiving for spouse”), and “low productive engagement group” (43.4%; low engagement in all productive activities). More detailed information about the 4 classes is shown in Supplementary Figure S1.

Individuals in the “family engagement group”, “working-family engagement group” and “society-family engagement group” were characterized by engagement in at least two productive activities. Given that compared with the non/low productive engagement group, productive engagement groups have similar impacts on the older adults’ mental health in previous studies, regardless of the number and type of the productive activity^20,27,28, ^this study merged “family engagement group”, “working-family engagement group” and “society-family engagement group” into a single productive engagement group to facilitate comparison with low productive engagement group. Notably, it is common practice to collapse data-driven LCA-derived patterns in research^44^. The subsequent network analysis was conducted based on two groups, namely the low productive engagement group and the productive engagement group. The proportion for the low productive engagement group and productive engagement group was 43.3% and 56.7% respectively.

### Network estimation of the overall participants

**Fig. 1:**
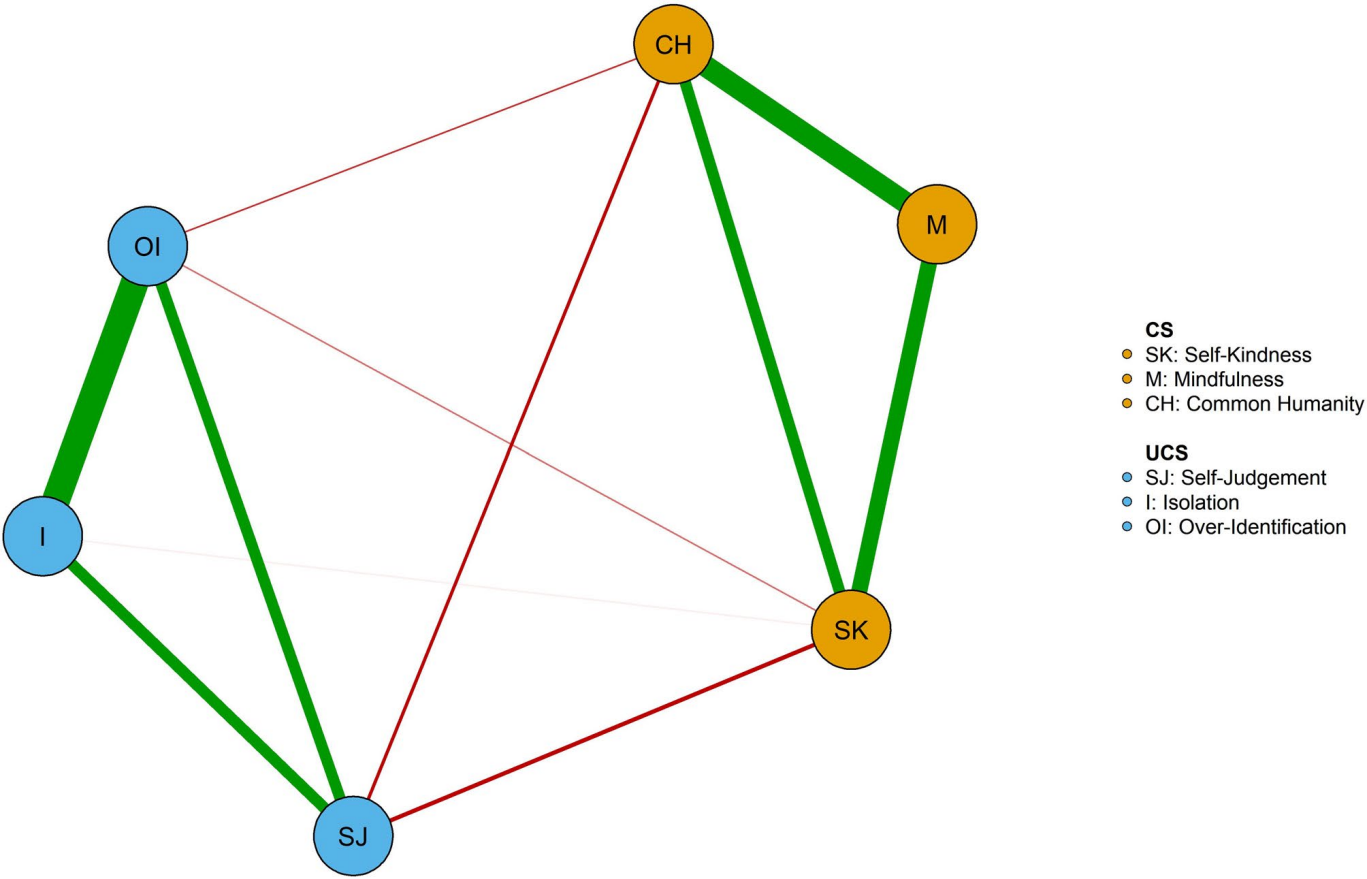
Network of self-compassion in the whole sample (*n* = 807). Green edges indicate positive associations between nodes; red edges indicate negative associations between nodes. Thicker edges between nodes represent stronger associations between nodes. Orange and blue nodes were defined as compassionate self-responding and uncompassionate self-responding. CS = Compassionate self-responding components, SK = *Self-Kindness*, M = *Mindfulness*, CH = *Common Humanity*, UCS = Uncompassionate self-responding components, SJ = *Self-Judgement*, I = *Isolation*, OI = *Over-Identification*. *Isolation* was identified as the core component due to the highest expected influence.

The self-compassion network of the whole sample is represented in Fig. 1. The network was dense (mean weight = 0.13), with 11 non-zero edges over 15 possible edges. As shown in Fig. 1, edges among all CS components and edges among all UCS components were positive. The edges between the CS components and UCS components were negative. The *mindfulness* was not associated with any UCS components. *Overidentification* and *isolation* (*r* = 0.61), *common humanity* and *mindfulness* (*r* = 0.47) had relatively high edge weight (see Supplementary Table S2).

**Fig. 2:**
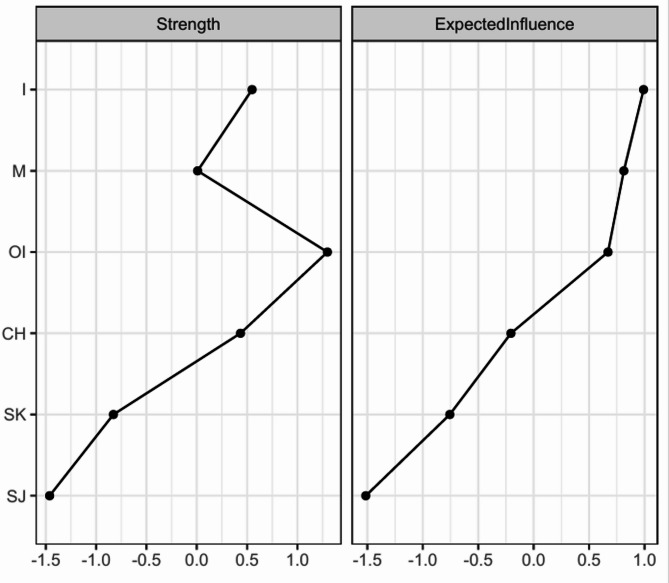
Centrality plot of the self-compassion network in the whole sample (*n* = 807). The nodes were ranked according to expected influence. I = *Isolation*, M = *Mindfulness*, OI = *Over-Identification*, CH = *Common Humanity*, SK = *Self-Kindness*, SJ = *Self-Judgement*. *Isolation* was identified as the core component due to the highest expected influence.

Figure 2 shows the expected influence centrality of each self-compassion component in the network. The stability of the expected influence was quantified using the CS-coefficient, and the result suggested an excellent level of stability of the expected influence (CS-coefficient = 0.75), i.e., after dropping up to 75% of the sample, the order of the nodes in expected influence was still correlated to the original sample (*r* > 0.7). As shown in Fig. 2, *isolation* was the central node with the highest expected influence.

#### Accuracy and stability estimation

The part of edge-weights with wide bootstrapped CIs indicated moderate precision for the edge weights (see Supplementary Figure S2). The stability of the centrality index examined using case-dropping bootstrapping is shown in Supplementary Figure S2.

### Network estimation for each group

**Fig. 3:**
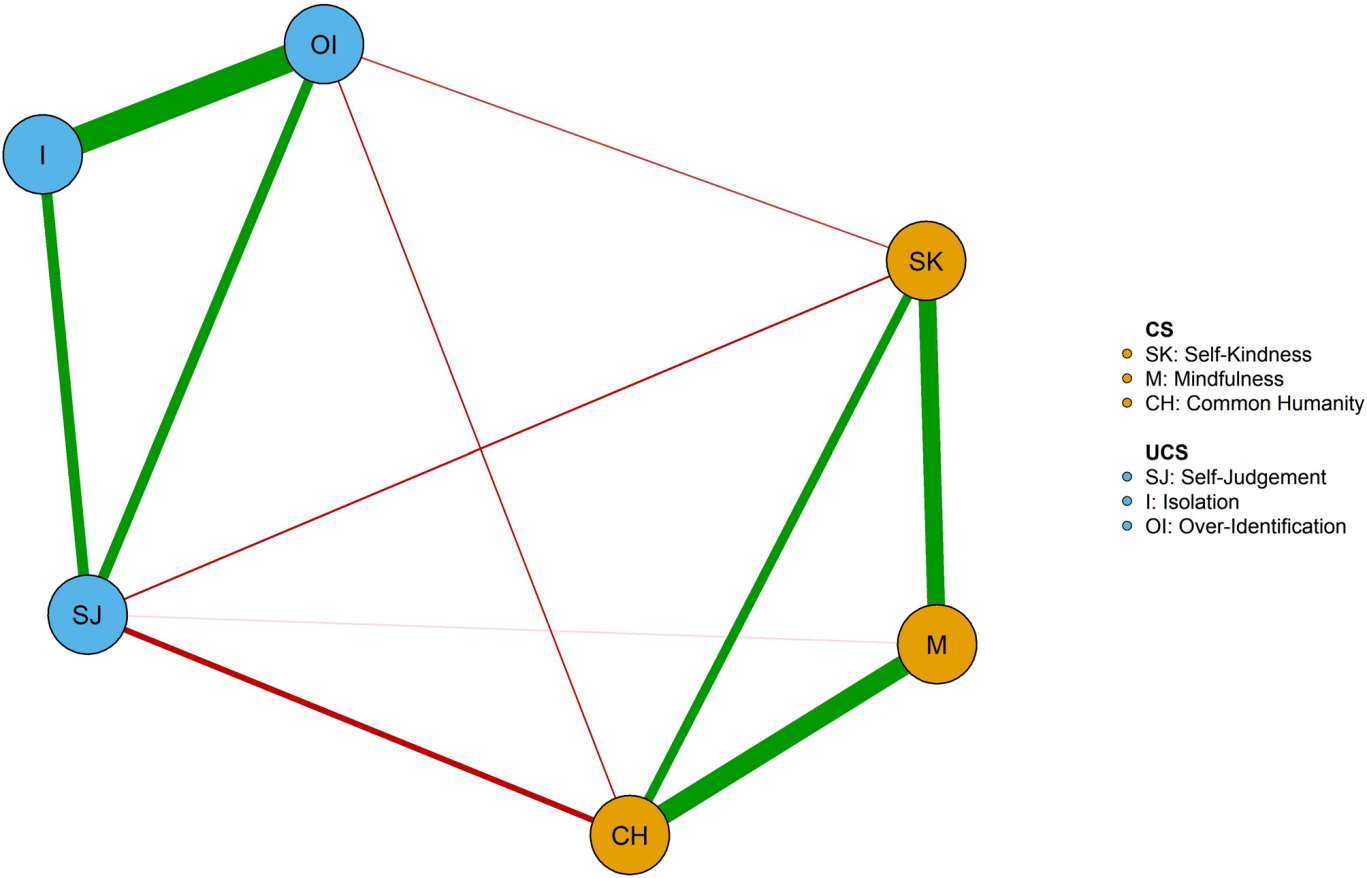
Network of self-compassion in the low productive engagement group (*n* = 350). Green edges indicate positive associations between nodes; red edges indicate negative associations between nodes. Thicker edges between nodes represent stronger associations between nodes. Orange and blue nodes were defined as compassionate self-responding and uncompassionate self-responding. CS = Compassionate self-responding components, SK = *Self-Kindness*, M = *Mindfulness*, CH = *Common Humanity*, UCS = Uncompassionate self-responding components, SJ = *Self-Judgement*, I = *Isolation*, OI = *Over-Identification*. *Isolation* was identified as the core component due to the highest expected influence.

The self-compassion network of the low productive engagement group is represented in Fig. 3. The network was dense (mean weight = 0.13), with 11 non-zero edges over 15 possible edges. As shown in Fig. 3, edges among all CS components and edges among all UCS components were positive. The edges between the CS components and UCS components were negative. The *isolation* was not associated with any CS components. *Overidentification* and *isolation* (*r* = 0.63), *common humanity* and *mindfulness* (*r* = 0.47) had relatively high edge weight (see Supplementary Table S3).

**Fig. 4:**
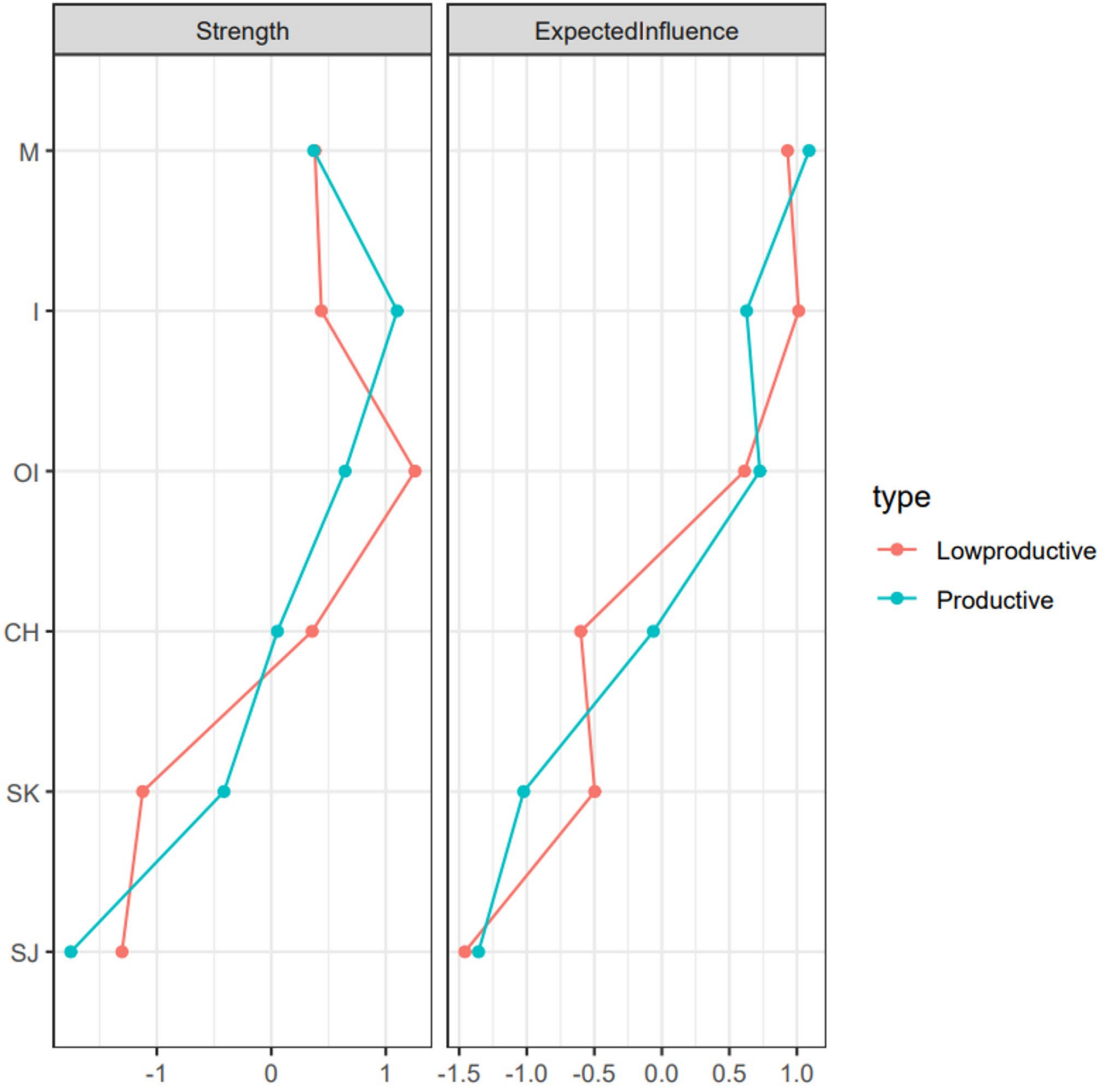
Centrality plot of the self-compassion network in the low productive engagement group (*n* = 350) and in the productive engagement group (*n* = 457). Pink value nodes indicate strength and expected infuence in the low productive engagement group, and blue value nodes indicate strength and expected infuence in the productive engagement group. Lowproductive = low productive engagement group, Productive = productive engagement group, M = *Mindfulness*, I = *Isolation*, OI = *Over-Identification*, CH = *Common Humanity*, SK = *Self-Kindness*, SJ = *Self-Judgement*. *Isolation* was identified as the core component in the low productive engagement group, while *mindfulness* was identified as the core component in the productive engagement group.

Figure 4 shows the expected influence centrality of each self-compassion component in the network of the low productive engagement group. The stability of the expected influence was quantified using the CS-coefficient, and the result suggested an excellent level of stability of the expected influence (CS-coefficient = 0.67), i.e., after dropping up to 67% of the sample, the order of the nodes in expected influence was still correlated to the original sample (*r* > 0.7). As shown in Fig. 4, *isolation* was the central node with the highest expected influence.

#### Accuracy and stability estimation of the self-compassion network in the low productive engagement group

The part of edge-weights with wide bootstrapped CIs indicated moderate precision for the edge weights (see Supplementary Figure S3). The stability of the centrality index examined using case-dropping bootstrapping is shown in Supplementary Figure S3.

**Fig. 5:**
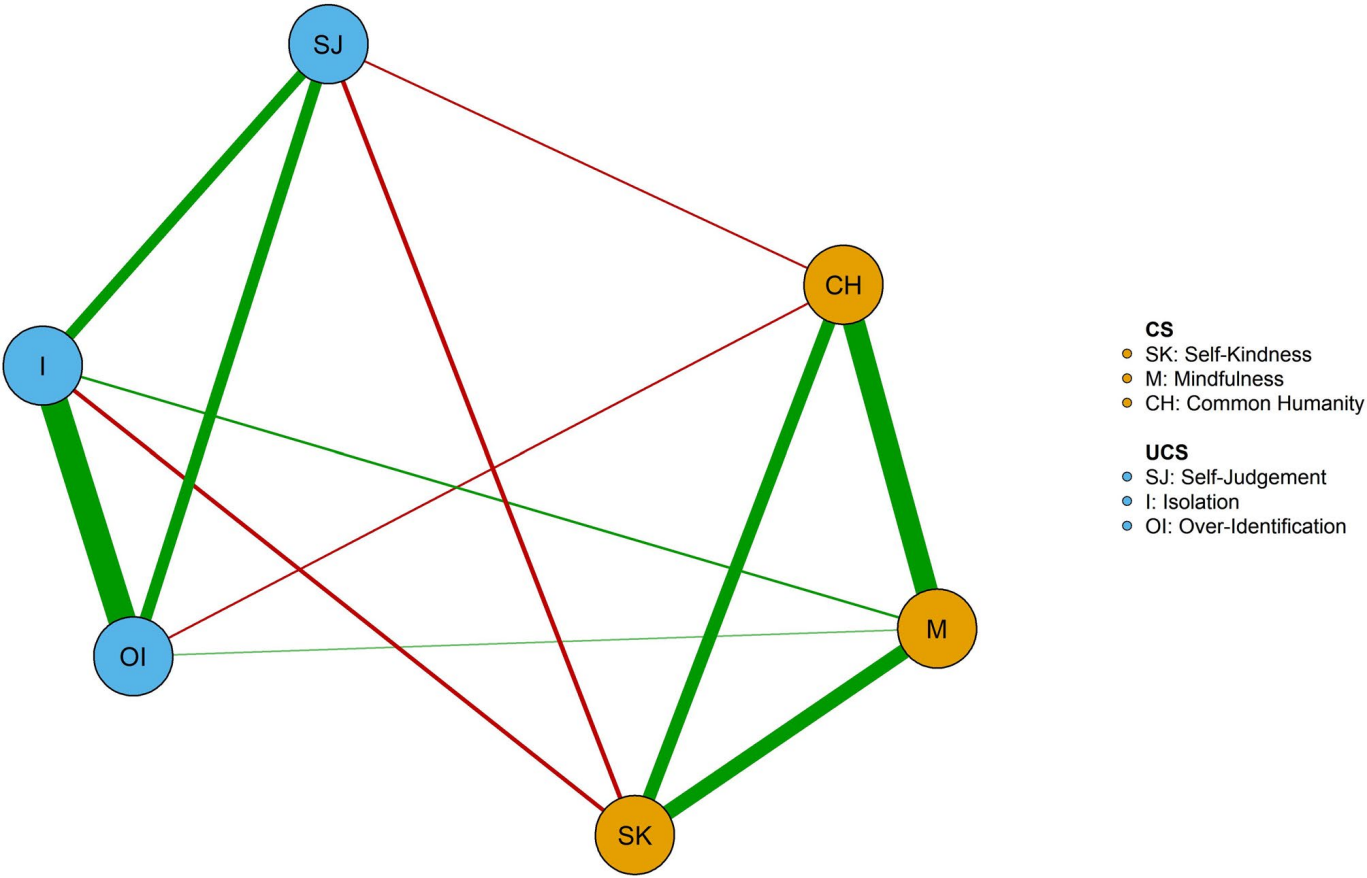
Network of self-compassion in the productive engagement group (*n* = 457). Green edges indicate positive associations between nodes; red edges indicate negative associations between nodes. Thicker edges between nodes represent stronger associations between nodes. Orange and blue nodes were defined as compassionate self-responding and uncompassionate self-responding. CS = Compassionate self-responding components, SK = *Self-Kindness*, M = *Mindfulness*, CH = *Common Humanity*, UCS = Uncompassionate self-responding components, SJ = *Self-Judgement*, I = *Isolation*, OI = *Over-Identification*. *Isolation* was identified as the core component due to the highest expected influence.

The self-compassion network of the productive engagement group is represented in Fig. 5. The network was dense (mean weight = 0.13), with 12 non-zero edges over 15 possible edges. As shown in Fig. 5 edges among all CS components and edges among all UCS components were positive. Some edges between CS components and UCS components were positive. *Overidentification* and *isolation* (*r* = 0.61), *common humanity* and *mindfulness* (*r* = 0.49) had relatively high edge weight (see Supplementary Table S4).

Figure 4 shows the expected influence centrality of each self-compassion component in the network of the productive engagement group. The stability of the expected influence was quantified using the CS-coefficient, and the result suggested an excellent level of stability of the expected influence (CS-coefficient = 0.75), i.e., after dropping up to 75% of the sample, the order of the nodes in expected influence was still correlated to the original sample (*r* > 0.7). As shown in Fig. 4, *mindfulness* was the central node with the highest expected influence.

#### Accuracy and stability Estimation of the self-compassion network in the productive engagement group

The part of edge-weights with wide bootstrapped CIs indicated moderate precision for the edge weights (see Supplementary Figure S4). The stability of the centrality index examined using case-dropping bootstrapping is shown in Supplementary Figure S4.

### Network comparison between different productive engagement groups

The network comparison tests show no significant differences in the network structure (*M* = 0.10, *p* = 0.68) and global strength (*low productive engagement group* = 2.52, *productive engagement group* = 2.67; *S* = 0.15, *p* = 0.26) of the two networks. The centrality invariance tests indicated no significant differences in the strength and the expected influence of nodes between the two networks.

However, comparisons indicated some specific connections between nodes that varied by productive engagement. The edge-weights were significantly different (*p* < 0.05) for 2 edges: *self-kindness* and *isolation*, *mindfulness* and *isolation*. Specifically, *self-kindness* was negatively associated with *isolation* only in the productive engagement group. Similarly, positive association between *mindfulness* and *isolation* was only found in the productive engagement group.

## Discussion

This study aimed to explore the network structure of self-compassion among older adults in Hong Kong, with a particular focus on its association with productive engagement patterns. Our findings fill the research gap in the internal structure of self-compassion in Chinese older adults and provide valuable insights into the potential difference in the self-compassion structure across productive engagement patterns, which further impacts the policy and interventions aimed at promoting the well-being of older adults.

The network analysis in the overall sample revealed positive relationships among three CS components and positive relationships among three UCS components. This finding is consistent with previous research, which highlighted the associations among self-compassion constructs^33,56^. Surprisingly, we did not identify a connection between *isolation* and its opposite construct *common humanity*, nor between *overidentification* with its opposite construct *mindfulness*. This result aligns with previous literature which also provides some support on the independence of CS and UCS components^56,57^rather than being on a single continuum in Chinese older adults. This finding may extend research about self-compassion structure in dialectical contexts^12^. A high level of certain CS component may not necessarily imply a low level of its opposite UCS in older adults and vice versa. For example, experiencing high isolation does not necessarily imply low common humanity, and similarly, high mindfulness does not always correspond to low overidentification. This self-compassion structure might derive from individuals in dialectical contexts being often encouraged to tolerant the co-occurrence of contradictory propositions^12^. Another possible explanation is the different self-compassion model in the Chinese context from the Western samples. Some researchers proposed that compared to the six-factor model of self-compassion, a novel four-factor model with three CS factors and one UCS factor (loaded by all items from three UCS components) might be more suitable for Chinese individuals^58–60^. This might also explain why there was a redundancy between *overidentification* and *isolation* in the pre-analysis of the current study. However, previous studies were conducted in Chinese adolescents or early and middle adult samples^58–60^. Therefore, we provided more insight into the specific inner structure of self-compassion in Chinese older adults, suggesting more explorations about the factor model of older adults’ self-compassion in future studies.

In addition, *isolation* was identified as the core component in the self-compassion network of older adults. Previous studies found that *isolation* was the strongest predictor of individuals’ depression among six self-compassion components^61–63^. *Isolation* might be more pervasive in older adults due to the increase in disability and the decrease in social integration^64,65^. Our findings may therefore provide a novel perspective for deteriorating mental health in Chinese older adults, in particular for the strong association between isolation and depression in previous research^55,61,63^. Social isolation is indeed known to heighten depression risk primarily through increased loneliness and negative social comparisons—processes already documented extensively in prior literature^61,66^. However, incorporating the self-compassion framework allows us to identify and elaborate upon specific psychological explanations and protective factors that traditional models of isolation and loneliness do not fully capture. Specifically, the self-compassion perspective highlights internal psychological responses—such as self-criticism, self-judgment—as critical elements mediating and moderating the relationship between isolation and depression. For instance, isolation may trigger heightened self-judgment or negative self-evaluation, especially within interdependent cultural contexts^67^thereby exacerbating depressive symptoms. By explicitly targeting these internal cognitive-emotional processes (e.g., reducing self-criticism). Self-compassion provides an actionable explanatory framework that traditional concepts like loneliness alone cannot fully articulate. Moreover, previous studies reported that isolation does not only directly associate with depression but also interacts indirectly with other self-compassion components (like self-judgment)^62^shaping complex pathways of psychological vulnerability. Therefore, self-compassion significantly extends our theoretical understanding by specifying internal processes. This deeper insight can inform more targeted, culturally sensitive interventions that directly address these underlying psychological processes, thus enhancing the conceptual clarity and applied relevance of our approach. This result might have some practical implications to support the implementation of compassion focused therapy which is a very important therapeutic techniques in reducing depression and loneliness in older adults^68–70^. In addition, the finding about the core role of isolation in older adults’ self-compassion network may suggest that self-compassion interventions for older adults can focus on reducing clients’ isolation, which could then change the whole structure of self-compassion and achieve a more effective improvement in their overall self-compassion.

Furthermore, this study explored the self-compassion network in older adults with different productive engagement patterns. The network of self-compassion was compared between two groups, one with low engagement at each productive activity (low productive engagement group) and the other one with high/moderate engagement at certain productive activities. Although the network structure and global strength of the two self-compassion networks were significantly identical, there were still differences in other features, such as the node centrality order. The *isolation* was found to have the strongest centrality in the low productive engagement group, while the *mindfulness* was identified to be the most central node in the productive engagement group. Our findings might provide some novel insights into the association between productive engagement and older adults’ well-being. Low engagement in productive activities (e.g., volunteering, educational courses) is associated with higher isolation in older adults, which plays a central role in the self-compassion network and possible linkage to negative outcomes, such as depression^6,71^. High engagement in productive activities is related to higher mindfulness, which is related to the rest of the self-compassion components and could link to positive outcomes in older adults, such as emotional well-being^72^. These results have significant practical implications, highlighting the importance of identifying older adults’ productive engagement patterns to effectively inform and personalize self-compassion interventions. By recognizing whether older adults exhibit high or low productive engagement helps to inform the central components within each group’s self-compassion network and tailor interventions accordingly. For example, older adults who demonstrate active productive engagement may particularly benefit from mindfulness-based workshops designed to enhance their mindful self-awareness. In contrast, older adults with lower productive engagement could benefit more from supportive interventions such as home visits or regular phone calls aimed at reducing isolation and fostering feelings of connection and self-kindness. Thus, accurately assessing productive engagement patterns is a crucial first step toward providing targeted and impactful self-compassion support for older adults.

The study also raised important theoretical implication on the competing effects of social roles through positive relationships between *mindfulness* and UCS components identified in the productive engagement group^22,23^. Considering the two competing effects from social roles of productive activities, mindfulness, overidentification, and isolation may all be associated at the same time by productive engagement. On the one hand, individuals who take part in meaningful activities may feel more mindful because of the social connections and sense of purpose of these activities^15,24^which could promote individuals’ thriving through several pathways, including emotional state (e.g., increase positive emotion), self-evaluations (e.g., self-acceptance), cognitive appraisals (e.g., positive appraisals)^73^. These three pathways align with *mindfulness*, which might be enhanced by social connectedness from productive engagement^24,74^. On the other hand, the same engagement can create stress if individuals feel overwhelmed by the demands of their social roles^23^. Increased stress is associated with *overidentification* and *isolation*^75,76^. Specifically, this stress can cause overidentification with these roles, where individuals may define themselves too much by their productivity or responsibilities. Such overidentification may lead to isolation, especially if individuals feel they cannot meet societal expectations or if they pull away from social interactions because of stress, suggesting that while productive engagement is helpful, it must be balanced to avoid negative psychological outcomes.

Our study hence may show the importance of promoting a healthy balance in social roles of productive activities in facilitating self-compassion for better mental health. When encouraging older people to engage in productive activities, it is necessary to avoid the potential side effects of role strain. While promoting productive activities, we need to pay attention to that older people might fail to meet the requirements and thus develop stress and low self-efficacy in some productive activities^77^. Specifically, regular interviews about role strain can be conducted with older adults to learn about their potential negative feelings (such as stress) during productive activities and then we can match older adults with appropriate demands and activities to reduce their potential negative feelings and allow them to benefit more from productive activities.

## Limitations and future directions

Despite meaningful insights into the structure of self-compassion and practical implications of well-being in older adults, there are also several limitations in the current study. Firstly, a cross-sectional design is applied in this study, which cannot identify the causality relationship of self-compassion components. Future studies can employ a longitudinal design to capture the dynamic relationships between self-compassion. Secondly, the sample in this study was recruited in Hong Kong Special Administrative Region (SAR) and does not represent the entirety of geographic areas in Asian societies. Future studies can explore the network structure of self-compassion among older adults residing in different geographic areas, to provide a more detailed picture of self-compassion and productive engagement in diverse sociocultural contexts. And due to the potential influence of Eastern dialectical contexts on the self-compassion structure^12, ^the generalization of our findings in other non-dialectical culture might be limited. Therefore, the exploration of older adults’ self-compassion in non-dialectical culture is required in future studies. Thirdly, the study data was collected in 2022, the middle of the COVID-19. Activities were limited in this period, which might influence older adults’ engagement in some productive activities, such as volunteering. It is possible that the result of this study may have underestimated the population of the productive engagement group in older adults and therefore needs to be replicated. Fourthly, although the SCS-SF was applied in Hong Kong older adults in previous research^39^ and good validity and reliability of SCS-SF were also reported in the current sample, research about the psychometrics of this scale in Hong Kong older adults is still limited, which needs more detailed exploration in future study. Fifthly, although this study focused on the difference in the self-compassion network between productive engagement groups and the low productive engagement group, we also tried to conduct and compare self-compassion networks among three productive engagement groups. However, the self-compassion network of “society-family engagement group” was unstable (CS-efficient = 0.28) due to small population size of this group. Future research can compare self-compassion networks in various productive engagement patterns to reveal potential differences.

## Conclusions

This research is the first to use the network technique to explore self-compassion structure and compare the self-compassion network between different productive engagement groups among older adults. CS components and UCS components might be separate constructs in Chinese older adults. *Isolation* is identified as the core component in the overall sample as well as older adults in the low productive engagement group, while *mindfulness* is identified as the core component in older adults in the productive engagement group. This suggests practical implications for aiming at older adults’ self-compassion, which is first identifying older adults’ productive engagement pattern and then focusing on isolation and mindfulness respectively to align with findings in the study.

## Electronic supplementary material

Below is the link to the electronic supplementary material.

Supplementary Material 1

## Data Availability

Data and study materials are available from the corresponding author on reasonable request.
