# Supplementary material for: The network structure of self-compassion in older adults with different productive engagement patterns
Source: Sci Rep. 2025 Jul 1;15:22321. doi: 10.1038/s41598-025-08157-1 (PMC12216479; doi:10.1038/s41598-025-08157-1)
Supplement: Supplementary file 1 — Supplementary Material 1 [file 41598_2025_8157_MOESM1_ESM.docx]

**The Network Structure of Self-compassion in Older Adults with Different Productive Engagement Patterns**

Huinan Hu^1^, Grand H.-L. Cheng^2^, Stephen Cheong Yu Chan^3^, Eddie S. K. Chong^1^, Peiyi Lu^1^, H.N. Cheung^1, *^

^1^ Department of Social Work and Social Administration, The University of Hong Kong, China

^2^ School of Arts and Social Sciences, Hong Kong Metropolitan University, China

^3^ Felizberta Lo Padilla Tong School of Social Sciences, Saint Francis University, China

**Supplementary Table S1.** Item names and descriptive statistics of items (*n* = 807)

|  | *M* | *SD* | *min* | *max* |
| --- | --- | --- | --- | --- |
| **Self-compassion components** |  |  |  |  |
| ***SK*** *(Self-kindness)* | 3.00 | 0.68 | 1.00 | 5.00 |
| ***M*** *(Mindfulness)* | 3.20 | 0.78 | 1.00 | 5.00 |
| ***CH*** *(Common humanity)* | 3.23 | 0.82 | 1.00 | 5.00 |
| ***SJ*** *(Self-judgement)* | 3.49 | 0.78 | 1.00 | 5.00 |
| ***I*** *(Isolation)* | 3.48 | 0.88 | 1.00 | 5.00 |
| ***OI*** *(Overidentification)* | 3.45 | 0.91 | 1.00 | 5.00 |
| **Productive activities (engagement)** | *n* | % |  |  |
| *Working* | 301 | 37.3% |  |  |
| *Formal volunteering* | 61 | 7.6% |  |  |
| *Informal volunteering* | 93 | 11.5% |  |  |
| *Learning* | 233 | 28.9% |  |  |
| *Care giving for spouse* | 389 | 48.2% |  |  |
| *Housework support for children* | 314 | 38.9% |  |  |
| *Caregiving for grandchildren* | 94 | 11.6% |  |  |
| *Caregiving for parents* | 160 | 19.8% |  |  |

**Supplementary Table S2**. Edge weight matrix of the self-compassion network in the whole sample (*n* = 807)

|  | SK | M | CH | SJ | I | OI |
| --- | --- | --- | --- | --- | --- | --- |
| SK | 0 |  |  |  |  |  |
| M | 0.36 | 0 |  |  |  |  |
| CH | 0.26 | 0.47 | 0 |  |  |  |
| SJ | -0.10 | 0 | -0.09 | 0 |  |  |
| I | -0.00 | 0 | 0 | 0.26 | 0 |  |
| OI | -0.03 | 0 | -0.04 | 0.25 | 0.61 | 0 |

**Supplementary Table S3**. Edge weight matrix of the self-compassion network in the low productive engagement group (*n* = 350)

|  | SK | M | CH | SJ | I | OI |
| --- | --- | --- | --- | --- | --- | --- |
| SK | 0 |  |  |  |  |  |
| M | 0.40 | 0 |  |  |  |  |
| CH | 0.22 | 0.47 | 0 |  |  |  |
| SJ | -0.06 | -0.01 | -0.14 | 0 |  |  |
| I | 0 | 0 | 0 | 0.25 | 0 |  |
| OI | -0.04 | 0 | -0.05 | 0.25 | 0.63 | 0 |

**Supplementary Table S4**. Edge weight matrix of the self-compassion network in the productive engagement group (*n* = 457)

|  | SK | M | CH | SJ | I | OI |
| --- | --- | --- | --- | --- | --- | --- |
| SK | 0 |  |  |  |  |  |
| M | 0.35 | 0 |  |  |  |  |
| CH | 0.28 | 0.49 | 0 |  |  |  |
| SJ | -0.11 | 0 | -0.06 | 0 |  |  |
| I | -0.10 | 0.07 | 0 | 0.25 | 0 |  |
| OI | 0 | 0.03 | -0.06 | 0.27 | 0.61 | 0 |

**
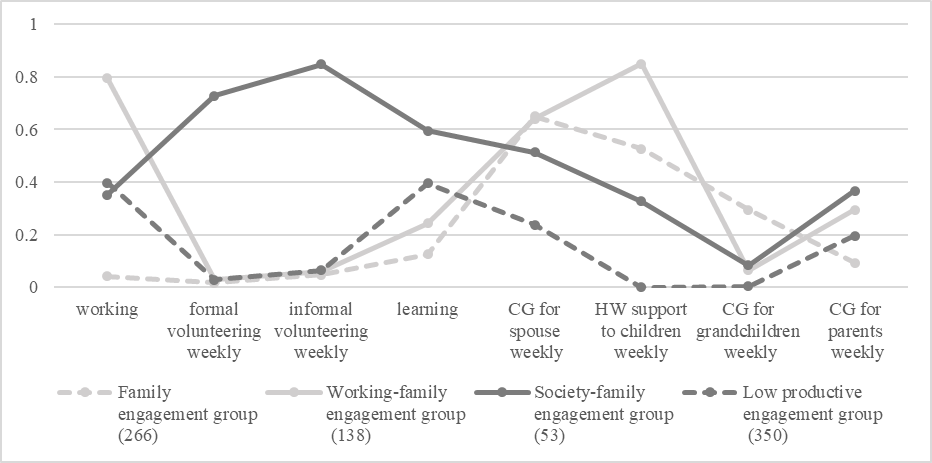
**

**Supplementary Figure S1.** Conditional item probability for the 4-class model of productive engagement (*n* = 807). CG = caregiving, HW = housework. Detailed information is presented in another prepared manuscript.


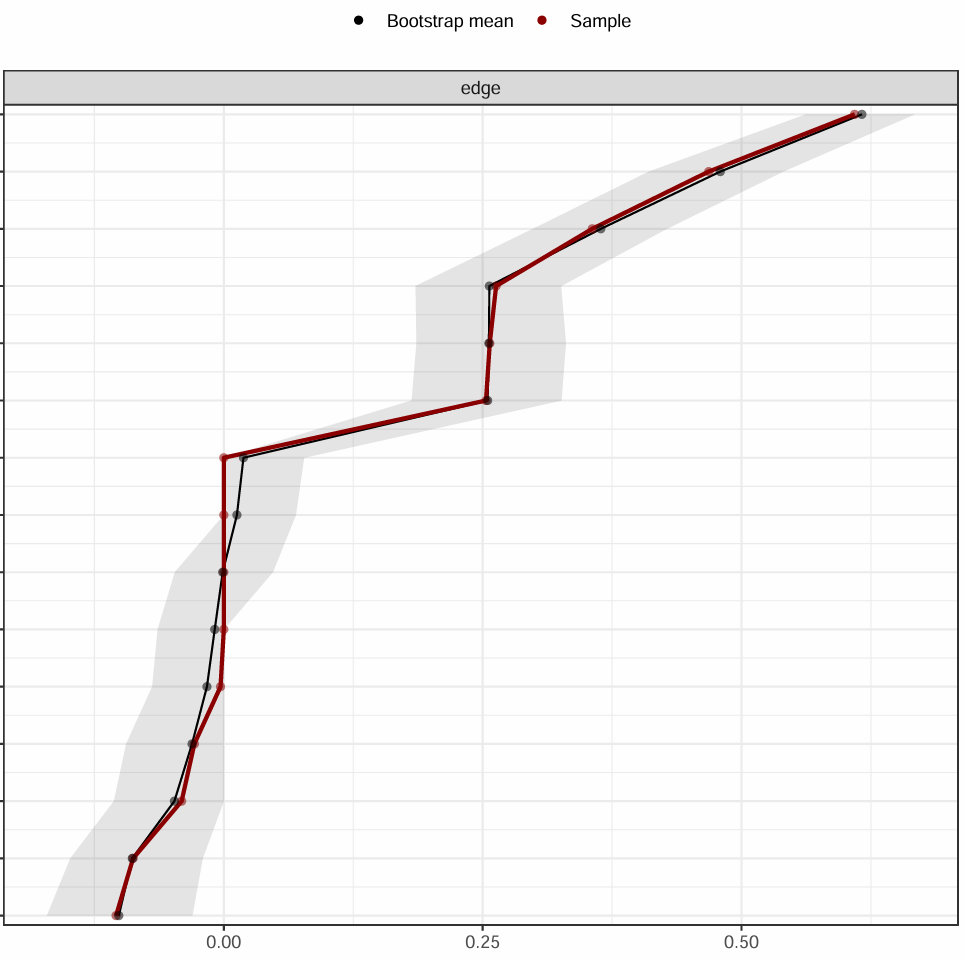
A
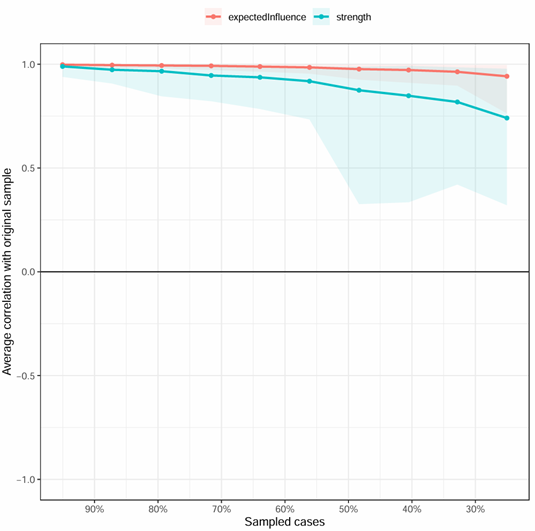
B

**Supplementary Figure S2.** (A) Bootstrapped confidence intervals of estimated edge-weights in the network of the whole sample and (B) Case-dropping bootstrap procedure for node centrality in the network of the whole sample (*n* = 807).


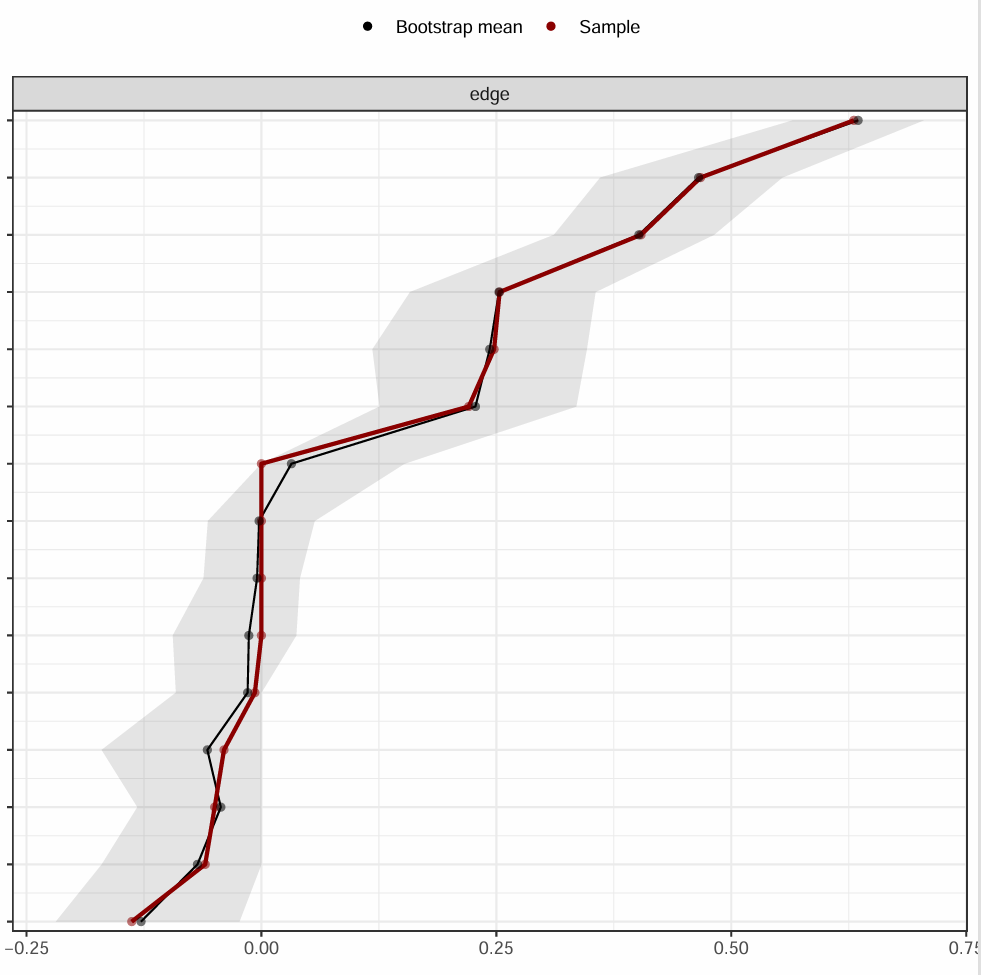
A
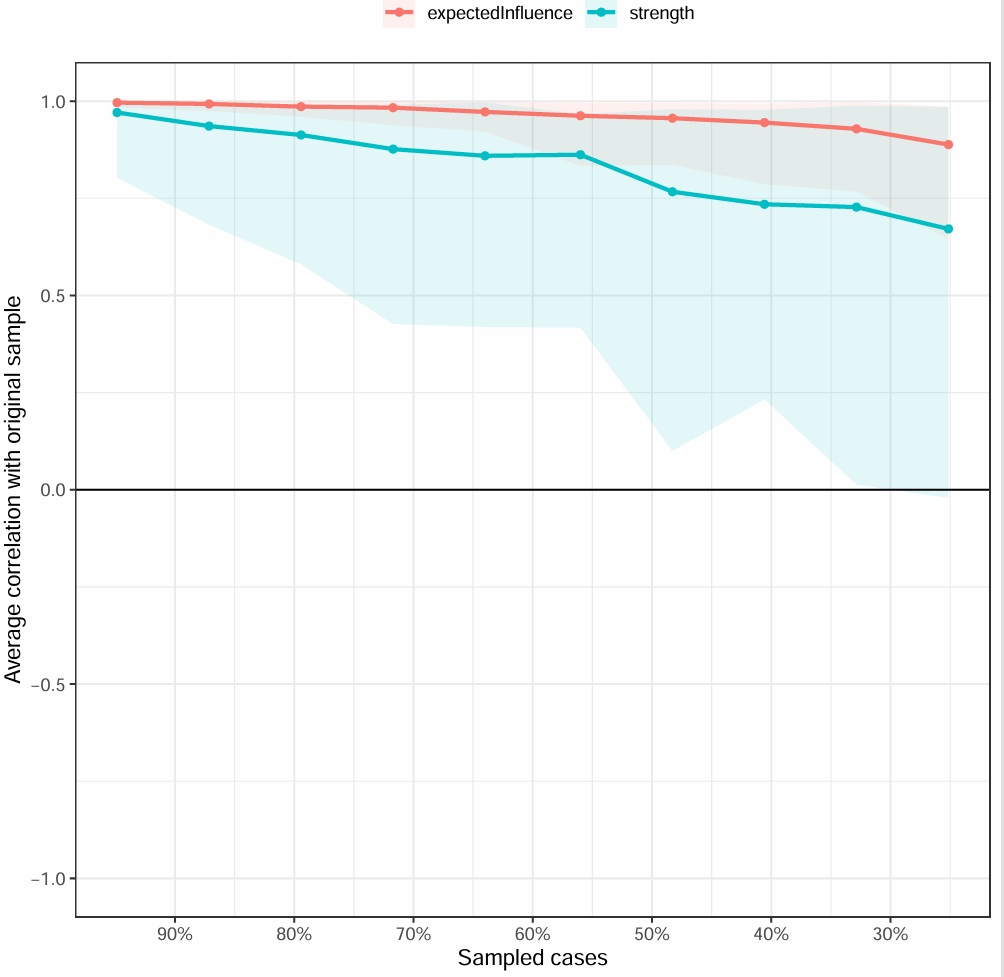
B

**Supplementary Figure S3.** (A) Bootstrapped confidence intervals of estimated edge-weights in the network of the low productive engagement group and (B) Case-dropping bootstrap procedure for node centrality in the network of the low productive engagement group (*n* = 350).


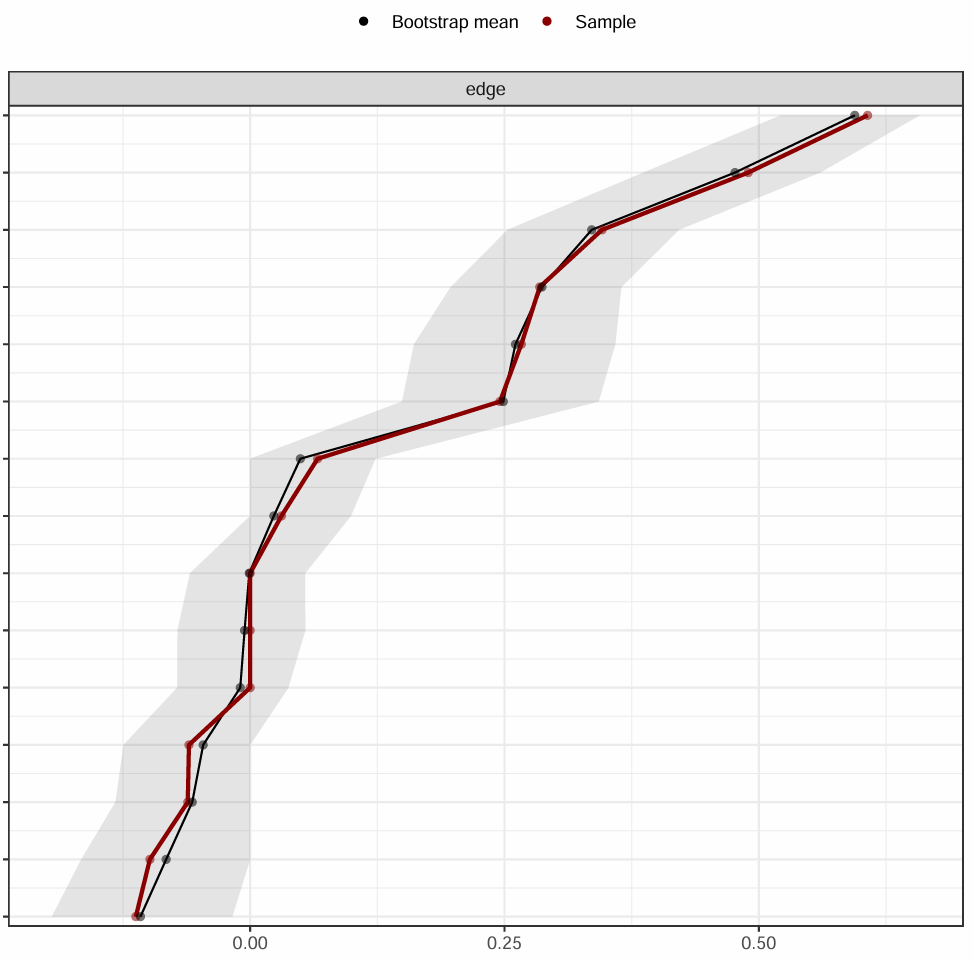
A
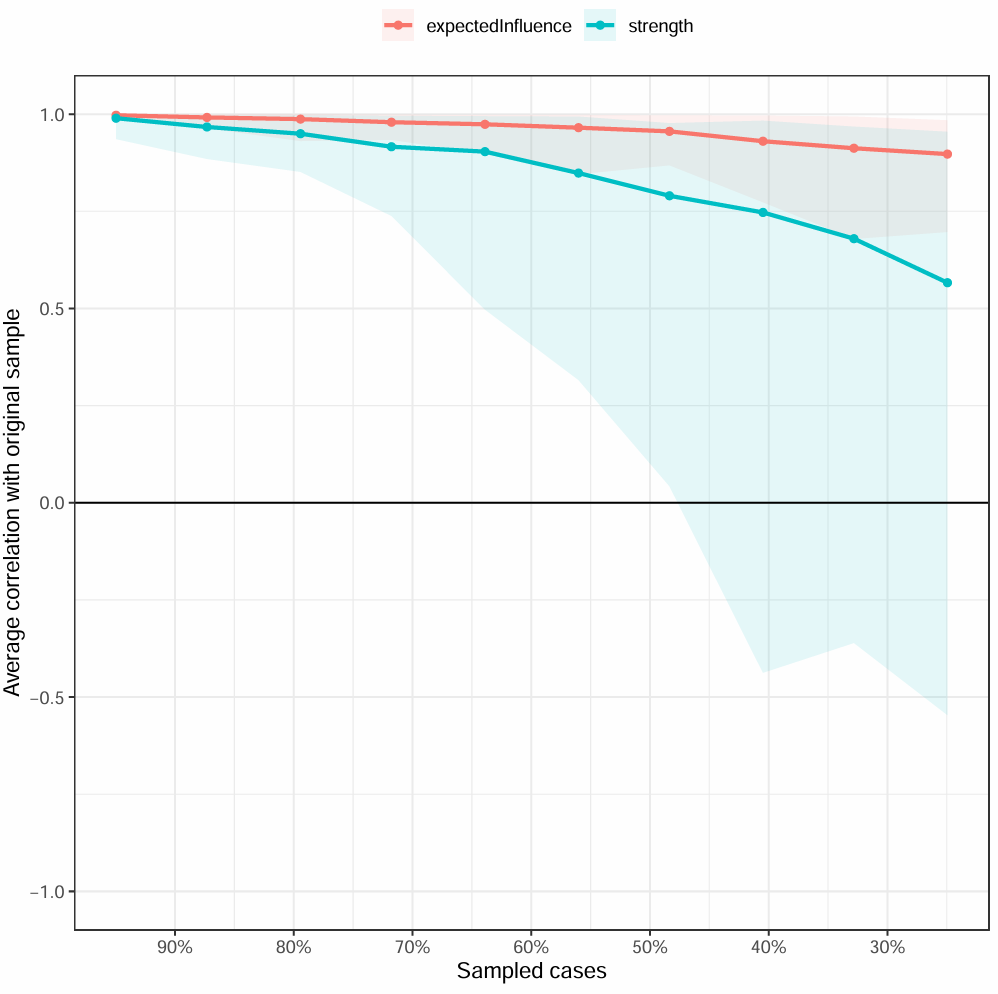
B

**Supplementary Figure S4.** (A) Bootstrapped confidence intervals of estimated edge-weights in the network of the productive engagement group and (B) Case-dropping bootstrap procedure for node centrality in the network of the productive engagement group (*n* = 457).
